# Origin of Petroleum

**Published:** 1867-05

**Authors:** 


*Origin of Petroleum.*—Berthelot, who has recently been working out the synthesis of hydrocarbons, has suggested a new theory to settle the puzzling question of the origin of petroleum. He has succeeded in forming a cetylene, a very rich hydrocarbon, ($C_4$, $H_2$,) by direct synthesis of its elements, and by a simple process in obtaining from it ethylene or olefiant gas. Without going into minute details, suffice it to say that acetylides are always formed when carbonic acid comes in contact with the alkaline metals at a high temperature. Now we know that the earth is everywhere impregnated with carbonic acid, and Danbree has recently shown good reason for believing that the terrestial mass contains melted alkaline metals in the interior. As earthy carbonates produce the same reactions with alkaline metals, we have all the conditions necessary for the generation of acetylides by simple mineral reactions. Now if these acetylides could be subjected to the action of steam, they would be decomposed and pure acetylene given off, if they could be cooled off and removed from the influence of hydrogen. But, as this cannot be, bitumen and tars are produced by the perpetual reaction of hydrogen, and at one of the stages, these reactions are capable of producing a series like the American petroleum.

Thus, we need not look for a quantity of vegetable matter, and a subterranean distilling apparatus and long undiscoverable streams leading to deep reservoirs, but can account for the phenomena by reactions which must needs be universal under the entire crust of the earth, so that petroleum may be found anywhere, if we bore deep enough.

